# Development of Gluten-Free Bread Production Technology with Enhanced Nutritional Value in the Context of Kazakhstan

**DOI:** 10.3390/foods13020271

**Published:** 2024-01-15

**Authors:** Nazira Utarova, Mukhtarbek Kakimov, Bożena Gajdzik, Radosław Wolniak, Ainur Nurtayeva, Saule Yeraliyeva, Michał Bembenek

**Affiliations:** 1 The Department of Food Technology and Processing Products, S.Seifullin Kazakh Agrotechnical Research University, Zhenis Avenue 62, Astana 010011, Kazakhstan; nazkon88@mail.ru (N.U.); ainur_78.05@mail.ru (A.N.); 2 Department of Industrial Informatics, Silesian University of Technology, 40-019 Katowice, Poland; bozena.gajdzik@polsl.pl; 3 Faculty of Organization and Management, Silesian University of Technology, 44-100 Gliwice, Poland; 4 The Department of Design and Technology, Korkyt Ata Kyzylorda University, 29A Aiteke Bi Str., Kyzylorda 120014, Kazakhstan; esg_1971@mail.ru; 5 Faculty of Mechanical Engineering and Robotics, AGH University of Krakow, A. Mickiewicza 30, 30-059 Krakow, Poland; bembenek@agh.edu.pl

**Keywords:** gluten-free bakery products, celiac disease, rice flour, corn flour, green buckwheat flour, plantain flour

## Abstract

This research aims to enhance the nutritional value of gluten-free bread by incorporating a diverse range of components, including additives with beneficial effects on human health, e.g., dietary fibers. The research was focused on improving the texture, taste, and nutritional content of gluten-free products by creating new recipes and including novel biological additives. The goal was to develop gluten-free bread with less than 3 ppm gluten content that can be eaten by people suffering from gluten sensitivity. The physical and chemical properties of gluten-free rice, corn, green buckwheat, chickpea, amaranth, and plantain flours were examined to understand their unique characteristics and the possibility of their mixing combination to achieve the desired results. Initially, nine recipes were prepared, and in survey research, four baking recipes were selected and tested. The composition of amino acids in the prepared gluten-free bread was determined. The variant made of corn, green buckwheat flour with plantain was found to be top-rated. Changes in the nutritional content of the new product were analyzed, and general regulations and nutritional values were identified. Experimental baking processes were carried out, leading to the successful formulation of gluten-free bread containing corn, green buckwheat, and plantain flour in a ratio of 40:40:20, meeting gluten-free requirements and demonstrating improved nutritional properties, as well as consumption properties, confirmed by surveys conducted on a group of consumers.

## 1. Introduction

Bread and baked goods are essential components of the daily diet. Therefore, the search for new ingredients to create innovative and natural gluten-free products [1] is ongoing, as the formulation of gluten-free products requires components that enhance dough flexibility, nutritional properties, and sensory attributes [2]. Given the increasing demand for gluten-free alternatives due to gluten intolerance and the numerous health benefits associated with gluten-free products [3], coupled with the exponential growth of the gluten-free food market in recent years [4], it is imperative to expand and diversify the production of gluten-free and functional foods [5]. Additionally, while individuals only require 10 milligrams of gluten per day, some gluten-free bread and baked goods contain more than the recommended amount, even exceeding 20 milligrams in the daily portion [6], underscoring the need to increase the variety of available gluten-free products [7]. Consequently, to broaden the range of pure gluten-free products, improvements in gluten-free bread and bun technologies, along with advancements in dietary therapy, have become pressing priorities.

The main raw materials used in the production of gluten-free bread products do not contain gluten, as other gluten-free flours are used to replace gluten-containing flours, resulting in a decrease in the nutritional value of the products [8]. This means that levels of dietary fiber, protein, B vitamins, and minerals (such as magnesium, zinc, iron, and copper) are significantly reduced [9]. Given the importance of nutritional value in making gluten-free bread, various methods to enhance and improve nutritional value are currently being considered and researched [10].

Another noteworthy aspect is the lack of products rich in natural, pure dietary fiber during the consumption of bread and baked goods. This is because the intake and levels of dietary fiber in daily foods are declining worldwide [11]. This is attributed to fewer individuals in the population consuming fiber-rich fruits, vegetables, and grains [12]. Dietary fiber is highly valued for its beneficial effects on gastrointestinal function, immunity, cardiovascular health, metabolism, and gut health [13].

The dietary fiber content of gluten-free bread, often a subject of interest, is scrutinized for its potential impact on digestive health and overall nutrition [14].

Malnutrition and food allergies are interconnected factors that can lead to gastric damage and disruption of the microbiota, contributing to various health conditions [15]. Among these health conditions is celiac disease, which has seen a steady increase in recent times [16]. This autoimmune disorder results in malabsorption in the stomach and progressive atrophy of the intestinal villi in genetically susceptible individuals [17]. Studies indicate that around 1% of the global population has celiac disease, and the only effective solution is to eliminate gluten-containing foods from the daily diet [18]. For individuals with celiac disease, adherence to a strict gluten-free diet is non-negotiable, as it prevents autoimmune reactions. Additionally, there is growing interest in the potential health benefits of a gluten-free diet for individuals with non-celiac gluten sensitivity, although more research is needed to establish the full extent of these benefits [19]. This text delves into the challenges of maintaining a balanced diet within the constraints of a gluten-free lifestyle, including considerations for nutrient deficiencies and overall dietary well-being [20].

Consuming gluten-free bread can have several health implications, both positive and potentially negative, depending on individual dietary needs and the specific product’s composition [21] (Table 1 and Table 2). For individuals diagnosed with celiac disease, the primary health implication of consuming gluten-free bread is the effective management of their condition.

Gluten-free bread may provide relief for individuals with non-celiac gluten sensitivity, a condition characterized by gastrointestinal symptoms and other discomfort when consuming gluten [22]. Some people with this sensitivity find that a gluten-free diet helps alleviate their symptoms, although more research is needed to fully understand this condition [23]. This type of bread may be lower in dietary fiber, iron, and B vitamins compared to traditional wheat-based bread. This is especially true if gluten-free bread is not fortified with these nutrients. Individuals on a gluten-free diet should be aware of potential nutrient deficiencies and consider alternatives and supplements as needed [24]. Some gluten-free bread products may be higher in calories and less satiating compared to their gluten-containing counterparts. This can have implications for weight management, and individuals should be mindful of portion sizes and overall calorie intake [25]. In some cases, a gluten-free diet, including gluten-free bread, can help improve gastrointestinal symptoms in individuals with gluten-related digestive issues. A reduction in gluten intake may alleviate symptoms such as bloating, abdominal discomfort, and diarrhea [26].

The glycemic index of gluten-free bread can vary depending on the ingredients used. For individuals with diabetes, this variation can impact blood sugar control. Choosing lower glycemic index gluten-free bread options is important for managing blood sugar levels [27]. Gluten-free bread, especially if not fortified with calcium, can contribute to reduced calcium intake. This may affect bone health, particularly in children, who require sufficient calcium for proper growth and development [28].

The technology for the production of gluten-free products has several problematic points. Because gluten-free products do not contain gluten, gluten-free bread has little porosity [41,42], and no rise [43]. To improve the technology of making gluten-free bread, as well as increase their nutritional values, many studies are being carried out to reduce the problems related to the consistency of the dough [44], decrease the difficulties in opening the dough [40], increase the low content of natural fibers [45,46], and trace elements [47].

Today, less attention is paid to technological approaches that change the properties of gluten-free bread, such as dough consistency [48], as well as extending the shelf life of gluten-free bread by expanding the ability to distribute gluten-free bread in a new, fresh form [2]. Gluten-free baked goods do not contain much fat and fiber, that is, they do not enrich [49]. In making gluten-free bread, it is recommended to use dietary fiber-rich ingredients to improve physical properties, organoleptic performance, and nutritional value [50]. Despite the growing market for gluten-free products, many gluten-free breads remain expensive [51] because they are often made with refined and expensive gluten-free flour or starch [52].

After studying the research on gluten-free bread, it was found that the consistency of the dough could be improved by:

- Adding several different compound flours when preparing gluten-free bread,
- Increasing nutritional value by adding an ingredient rich in dietary fiber,
- Increasing the amount of dietary fiber, as well as increasing vitamin content, and reducing the price of gluten-free bread by using Central Asia raw materials instead of imported ones.

As a result of this research, a beneficial natural product, gluten-free clean bread, and baked goods have been developed and tested in surveys in terms of taste and consistency to address and prevent gastrointestinal and allergic diseases, including celiac disease, and promote overall human health. Additionally, nutritional value has been enhanced, and clean food consumption with high nutritional quality is encouraged. These innovative works can contribute to the improvement of human health through the provision of gluten-free, clean, and nutritious bread and flour products.

## 2. Materials and Methods

### 2.1. Materials

To make gluten-free bread enriched with dietary fiber, vitamins, macro and microelements, the components of various gluten-free compound flours were studied, and their percentage rates were determined. Gluten-free rice, corn, green buckwheat, chickpea, amaranth, and plantain flour (Al I KS Firm LLP, Kazakhstan, Astana) were used in the development of gluten-free bread.

As mentioned before, many studies have focused on the development of recipes and technologies for gluten-free baked goods based on corn and rice flour. Among them, a technique proposing the technology of gluten-free bread based on corn starch with the addition of 30% rice and 40% buckwheat flour instead of starch was introduced [53]. In addition, in the process of making gluten-free bread, it has been observed that adding 17.14% plantain to the recipe has the effect of prolonging the texture, appearance, texture, and suitability of gluten-free bread [54].

For this reason, based on these studies, studying the recipes for making gluten-free bread revealed that when preparing gluten-free bread, the main percentage of flour should range from 40 to 50%, with plantain accounting for 10–20%, whereas in the process of producing ready-made gluten-free bread, bread with 40% corn, green buckwheat flour, and a 20% dose of plantain showed the best organoleptic indicators. Adding 17.14% of plantain to the recipe further increased the shelf life of the gluten-free bread by 20%, so the optimal ratio from the above characteristics was chosen so as not to receive dissatisfaction due to changes in taste, color, and aroma. 

In Table 3, the organoleptic and physico-chemical indicators and their percentage contents of the used flours are presented. These were chosen to improve the quality of gluten-free bread, extend its shelf life, and expand the range of bakery products.

### 2.2. Methods

#### 2.2.1. Sensory Evaluation of Flour

To assess the quality of gluten-free flours, a sensory evaluation was conducted. 20 trained testers evaluated the appearance, smell, color, and taste of different flour samples. The aim was to identify flours with desirable organoleptic properties suitable for gluten-free bread production. During the organoleptic assessment, indicators such as type, color, smell, and taste of flour were determined. The assessment was carried out on a ten-point system, where 1 is the lowest rating and 10 is the highest. The values were averaged from the ratings of all respondents. Organoleptic indicators of flour were determined according to GOST 27558-87 “Flour and bran. Methods for determination of color, odor, taste, and crunch” [60].

First, to determine the color of the flour, a plate with flour samples was immersed in a container of water at room temperature in an inclined position (30–45°), and removed from the water after stopping the release of air bubbles. Then, the color of the flour was determined. To determine the smell of the flour, 20 g of flour was poured onto clean paper and warmed by inhaling, installing the smell. The taste of flour was determined by chewing.

#### 2.2.2. Physicochemical Analysis of Flour

Physicochemical properties of the gluten-free flours were analyzed. Parameters such as moisture content, fiber content, bulk density, and ash content were measured. 

Flour moisture content was measured based on GOST 9404-88 “The flour and bran. Method of moisture content determination” [61]. The moisture content of the flour was determined by drying a flour sample (5–10 g) in a drying cabinet at a temperature of 105 °C until a constant weight was achieved. The percentage of moisture in the flour was calculated by the weight difference before and after drying.

Bulk density was measured in cylindrical test tubes of 50 mL volume. First, the tubes was weighed, and the flour sample was filled by constant tapping until the volume no longer changed. The difference in weight was determined, and bulk density was calculated in kilograms per cubic meter (kg/m^3^).

Flour ash content was determined based on GOST 27494-2016 “Flour and bran. Methods for determination of ash content” [62]. A flour sample of around 20–30 g was transferred to a glass plate and mixed with two flat scoops. Then, it was pressed down with another glass to obtain a thickness of 3 to 4 mm. After removing the upper glass, two samples, each weighing 1.5–2.0 g, were taken from at least ten different places. These samples were placed in two crucibles, pre-calcined to a constant mass and cooled in a desiccator at ambient temperature. The suspended crucibles with samples were placed at the door of a muffle furnace PLF 100-3 (Erkaya, Ankara, Turkey), heated to 400–500 °C (dark red heat), after which the samples were charred, preventing the ignition of dry distillation products. After the separation of dry distillation, the crucibles were pushed into the muffle furnace, and the door was closed. The muffle furnace was then heated to 600–900 °C (bright red heat). The tests were carried out until the black particles completely disappeared, and the ash color turned white or slightly grayish. The laboratory scales Demcom DL-63 (Demcom, Milan, Italy) were used for the test.

The fiber content in the flour was determined according to GOST 13496.2-91 “Fodders, mixed fodders, and mixed fodder raw material. Method of determination of raw cellular tissue” [63]. A 1 g flour sample was placed in a glass with a capacity of 300–400 cm^3^. Then, 100 cm^3^ of sulfuric acid, preheated to boiling, was poured into the glass. The liquid level in the glass was marked. The contents of the glass were stirred with a glass stick and softly boiled on a tile for 10 min, starting from the beginning of boiling, with occasional stirring. Then, the glass was removed from the tile, and the stuck particles were washed off the walls with water with the help of a glass stick, ensuring that the liquid level in the glass reached the mark but did not exceed it. Then, 28 cm^3^ of potassium hydroxide solution was poured, stirred with a stick, and boiled again for 10 min. After boiling, the precipitate settled, and the solution was filtered by decantation through a pre-dried paper filter. Then, the precipitate from the glass was transferred to the filter with a solution of hydrochloric acid, and the filter was washed twice with the same solution, using 20 cm^3^. Then, the filter and fiber were washed to a neutral reaction (3–4 times) with hot water, and about 20 cm^3^ of alcohol and 20 cm^3^ of diethyl ether. After that, the filter was placed on a Buchner funnel (Kartell, Suponevo, Russia) inserted through a rubber stopper into a Bunsen flask (Pyrex, Klin, Russia). Filtration was carried out under vacuum. The filter with the washed fiber was dried and weighed.

#### 2.2.3. Dough Preparation and Bread Baking Process

Different formulations of gluten-free dough were prepared by combining various flours and other ingredients. The optimal amount of raw materials was determined based on the ratios of the samples prepared is presented in Table 3. The recipe for gluten-free bread is shown in Table 4.

##### The Process of Baking Bread

First, the raw materials were sifted, weighed, and prepared. The temperature and amount of water were calculated at 49.0% ± 1, taking into account the temperature and humidity of the dough. The dry substances: 160 g of the flours (in the shares presented in Table 3), 7 g of pour yeast, 5 g of salt, and 10 g sugar, were placed in the dough kneader KitchenAiD Professional K45SS mixer (KitchenAiD Europa Inc., Brussels, Belgium). Then, 180–200 g of water was added (depending on the moisture of the flour), and everything was mixed together at the lowest speed for 5 min, until the dough came to a uniform shape. The kneaded dough was removed and placed on the thermostat TSO-160 (MIZ-MA, Belgorod-Dnestrovsky, Ukraine) at 28 °C and 75% relative humidity for 60 min to ferment. The kneaded dough was divided into pieces weighing 350 g. The bread was made into a round shape and put in molds greased with sunflower oil, flattened a little, and then placed on the thermostat TSO-160 (MIZ-MA, Belgorod-Dnestrovsky, Ukraine) at 28 °C for 60 min. The surface of the bread blanks was moistened with water, and the molds were placed in the oven. After placing the bread in the oven, water was poured into the filling container inside the oven, allowing the bread to be soft and keep the outer surface well-baked. The breads were baked in the Revent 7121 oven (Revent, Upplands-Väsby, Sweden) at 190 °C for 25 min, then at 170 °C for 10 min. The baked goods were taken out of the oven and cooled at an ambient temperature of 25 °C for 2 h.

#### 2.2.4. Preliminary Selection of Loaves for Further Testing

The 9 different types of bread loaves, baked and cooled, were pretested. The tasting was conducted according to the appearance, color, smell, porosity, and taste of the bread. 18 people took part in the survey. The tasting evaluation of the 9 different types of bread was carried out on a 5-point scale (5—excellent, 4—good, 3—satisfactory, 2—bad, 1—very bad), and each participant entered their results on the tasting sheets. The test results were averaged. Four types of bread with the highest average rating obtained in the surveys were selected for further research. The selected loaves were organoleptically assessed.

#### 2.2.5. The Determination of Gluten Content in the Bread

To ensure the gluten content remained below the required threshold (<3 ppm), gluten analysis was conducted on the prepared bread. To accurately measure gluten levels, specialized testing methods such as Ridascreen Gliadin (R-Biopharm AG, Darmstadt, Germany); Veratox for Gliadin R5 (Neogen, Lansing, Michigan, USA); and AgraQuant Gluten G12 (Romer Labs, Getzersdorf, Austria) were used. All values were designated as mean ± standard deviation (*n* = 3).

The gluten content in the gluten-free bread product was analyzed using the gliadin test Ridascreen (R-Biopharm AG, Darmstadt, Germany), which was also used by Ja Myung Yu [64].

#### 2.2.6. Analysis of Amino Acids and Vitamins Composition of the Bread

The nutritional composition of the gluten-free bread was analyzed to determine its amino acid profile and overall nutritional value. Essential amino acids, vitamins, and other nutrients were evaluated to confirm the bread’s nutritional adequacy. The amount of amino acids and vitamins contained in gluten-free bread were measured with a capillary electrophoresis system Capel-105 (Tacticom LLP, Minsk, Belarus). It was decided that only bread with the best recipe and parameters selected by the respondents would be tested.

The determination of the content of amino acids and vitamins in gluten-free bakery products was carried out using capillary electrophoresis, according to method M 04-41-2005. Capillary electrophoresis is currently one of the most promising and highly effective methods for separating and analyzing complex mixtures into constituent components, with advantages such as extremely low consumption of reagents and solvents, analysis speed, and the minimum volume of the analyzed sample being dosed. To determine the vitamins, 1–2 g of the sample was measured, and a solution of sodium boric acid and sodium sulfide in a ratio of 3:2 was poured over it, isolating the vitamins. Whisk at room temperature for 15 min. After filtering it out, we lowered the filtrate into the “Droplet”. The length of the dropper is 75 cm, and the diameter is 50 microns.

#### 2.2.7. Determining the Color of Bread on the CIELab (L*a*b*) Scale

Determination of the color of the bread on the CIELab scale was carried out in Adobe Photoshop CS3 (Adobe Systems Incorporated, San Hose, CA, USA) based on photos of the bread cross-section taken with the camera of the iPhone 12 MGJC3RM/A (Apple Inc., Cupertino, CA, USA). Due to the heterogeneous structure of the bread, in order to average the color of the bread over the entire cross-section, an average blur filter was used before taking the color sample.

### 2.3. Statistical Analysis

To analyze the relationships between individual analyzed types for both flour and bread, a statistical method of cluster analysis was employed. The calculations were performed using Statistica software version 13.3.

The tree-based cluster analysis statistical method employed in this study utilizes a hierarchical clustering technique, visually represented as a dendrogram or tree-shaped structure. In this method, objects are grouped into hierarchical clusters, wherein each cluster comprises the most similar objects, and higher-level clusters encapsulate those from lower levels. Following an agglomerative approach, the clustering begins with individual objects and progressively merges the closest pairs iteratively, forming increasingly larger clusters until all objects are within a unified cluster [65,66].

## 3. Results

### 3.1. Study of Organoleptic Indicators of Flour

The results of organoleptic indicators of flours are shown in Table 5. The results meet the general standards [65,66,67] for all flours. 

From the data in Table 5, it can be seen that green buckwheat, corn, and plantain flour showed high scores for all organoleptic indicators. Other types of flour also received pretty good scores. The overall results, in terms of points for all types of flour, show that all types of flour meet the general standard for further use in making bread.

**Figure 1:**
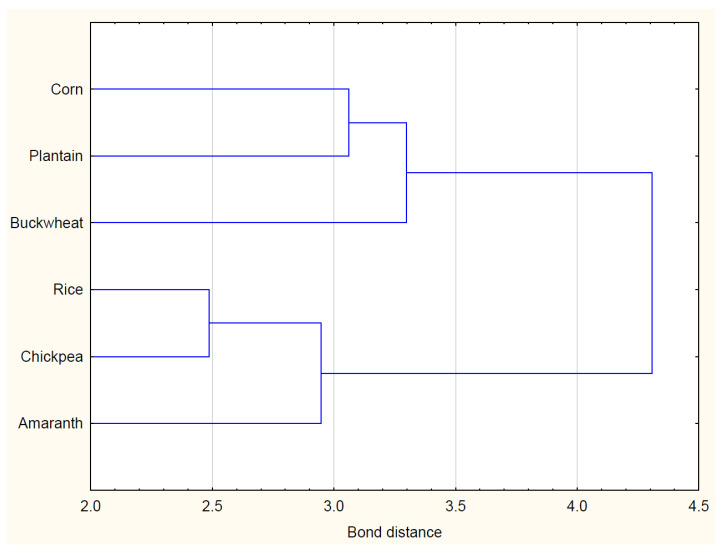
Cluster analysis for the flour types.

In the case of applying cluster analysis to the flour, the corresponding figure presented in the results of the analysis is shown in Figure 1. Assuming a distance of no more than 3.5, two groups can be distinguished:

- First group—corn, plantain, and buckwheat. These three flours (corn, plantain, and buckwheat) share similarities in certain characteristics that make them part of the same cluster. The closeness or similarity in their composition or properties led to their grouping.
- Second group—rice chickpea, amaranth. Rice, chickpea, and amaranth form a separate cluster distinct from the first group. This indicates that these three flours have similarities among themselves but are distinct from the flours in the first group.

The cluster analysis in the paper reveals distinct groupings of flour types based on certain similarities. The identified clusters include one with corn, plantain, and buckwheat, and another with rice, chickpea, and amaranth. These clusters may be indicative of underlying compositional or functional attributes. These flour clusters appear to align with the organoleptic evaluations of bread samples. The paper emphasizes the successful development of gluten-free bread, with specific combinations of flours leading to optimal sensory characteristics. For instance, bread made from a combination of corn, green buckwheat flour, and plantain, falling within the same cluster, exhibited the best overall indicators.

The gluten content analysis and amino acid compositions may also be influenced by these flour clusters. Certain clusters might predominantly contain gluten-free flours, contributing to the overall success of achieving gluten-free bread with desirable qualities. The vitamin composition of the gluten-free bread is discussed in relation to flour types. The cluster analysis likely guided decisions on flour combinations, contributing to the improved nutritional profile of the bread. Notably, the patented method for gluten-free bread production involves a unique blend of flours, and these flour clusters could have played a role in its development.

The practical implications of the cluster analysis are evident in the successful technology development for gluten-free bread. The identified flour clusters provide a foundation for future research, aligning with the paper’s suggestion to explore the interplay between different compositions of gluten-free flours.

### 3.2. Study of the Physico-Chemical Composition of Flour

The physicochemical composition of flour, including moisture content, ash content, fiber content, and bulk density, is shown in Table 6.

From the data in Table 6, it can be seen that rice flour has the highest moisture content, and plantain flour has the lowest. The fiber content is between 3.5% in chickpea flour to 11.9% in plantain flour. The highest ash content is found in plantain flour. According to the results of the analysis of physico-chemical indicators of corn flour, the moisture content of corn flour was 8.23%, ash content was 0.3060%, and fiber was 7.3%. The ash content in the bread, according to GOST 27494-2016 “Flour and bran. Methods for the determination of ash content”, can be up 0.9%. Comparing the results with Gwirtz et al. [70], it can be seen that the physicochemical indicators of corn flour meet all standards. According to the results of the analysis of physicochemical indicators of green buckwheat flour, the moisture content was 7.85%, ash content was 0.4264%, and fiber was 10.6%. The results are similar to Mohojan et al. [71] and generally meet the basic requirements. According to the results of the analysis of physicochemical indicators of rice flour, the moisture content of rice flour was 9.11%, ash content was 0.3689%, and fiber was 2.4%. The results of the study are similar to those of other scientists [72]. According to the results of the analysis of physicochemical indicators of chickpea flour, the moisture content of chickpea flour was 7.91%, ash content was 0.3005%, and fiber was 3.5%. The research results are consistent with the results obtained by Kotsiou et al. [73]. According to the results of the analysis of physicochemical indicators of amaranth flour, the moisture content of amaranth flour was 8.32%, ash content was 0.4120%, and fiber was 4.7%. When comparing the research work of Gebreil et al., it can be seen that the physicochemical indicators of amaranth flour meet all standards [74]. According to the results of the analysis of physicochemical indicators of plantain, the moisture content of plantain was 7.18%, ash content was 0.8295%, and fiber was 11.9%.

### 3.3. Preliminary Selection of Loaves for Further Testing

The tasting evaluation of 9 different types of bread is presented in Table 7. All samples presented to the tasting commission had high-quality indicators. According to the results of the tasting, out of 18 people who took part in the general tasting, bread samples No. 3 and 5 were rated the highest by appearance, porosity, taste, bread samples No. 2 and No. 5 by color, and bread samples, No. 5, No. 9, and No. 3 by smell. The highest average scores—4.39; 4.17; 4.04; 4.01—were obtained by samples No. 1, 3, 5, and 9. In the fifth sample, the tasters noted the pronounced pleasant taste and aroma of buckwheat, and in the third—the rich color of the crumb. According to the results of the tasting, loaves No. 1, 3, 5, and 9 were selected for further research.

**Figure 2:**
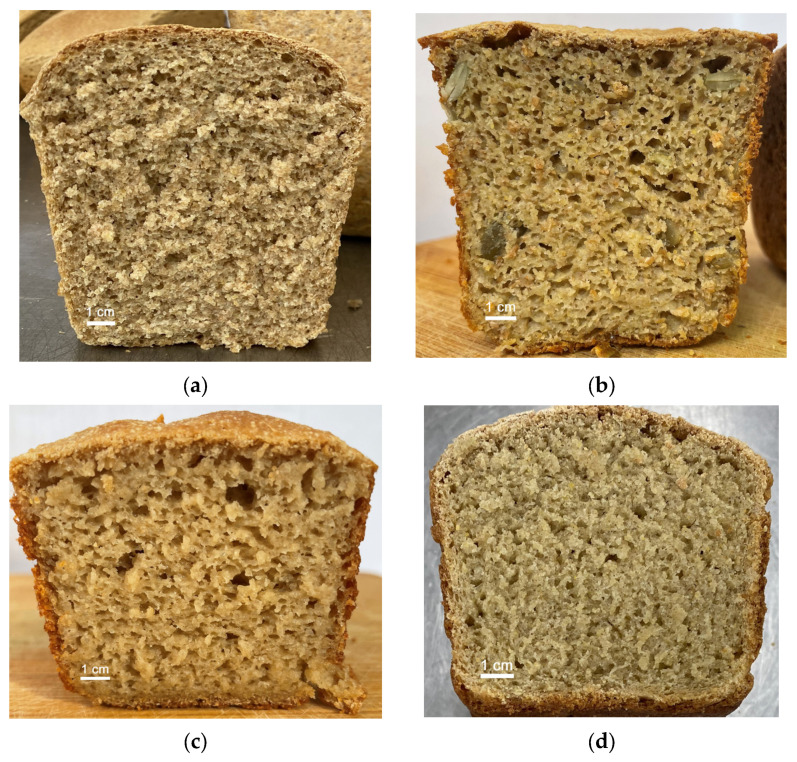
Evaluation of porosity and the state of softness during organoleptic evaluation of bread samples: (**a**) rice, green buckwheat, and plantain flour in a ratio of 50:40:10 (No. 1); (**b**) rice, green buckwheat, and plantain flour in a ratio of 40:40:20 (No. 3); (**c**) corn, green buckwheat, and plantain flour in a ratio of 40:40:20 (No. 5); (**d**) corn, green buckwheat flour, and plantain in a ratio of 50:40:10 (No. 9).

An organoleptic assessment of the state of softness and porosity of bread samples baked in different ratios is shown in Figure 2 and Table 8.

**Figure 3:**
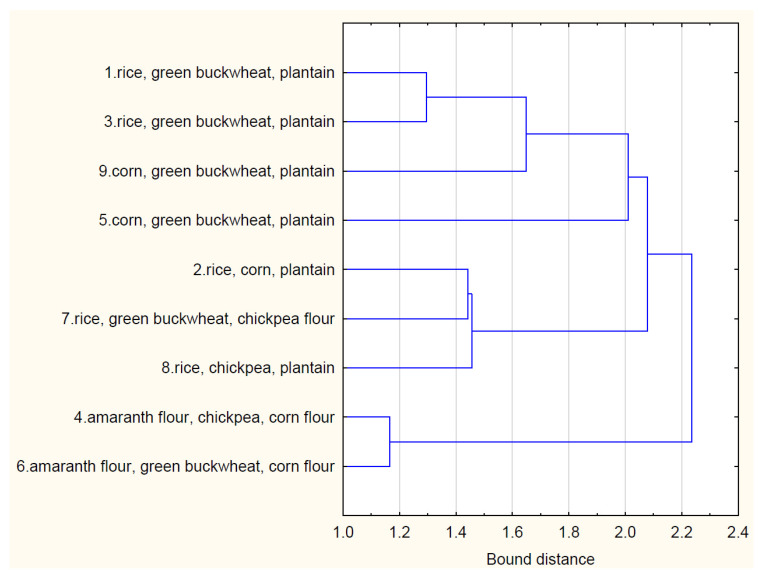
Cluster analysis for the bread types.

In the case of applying cluster analysis to bread, the results of statistical analyses have been presented in Figure 3. Assuming a distance of no more than 1.8, four groups can be distinguished:

- First group—1 rice, green buckwheat, plantain; 3—rice green buckwheat, plantain; 9—corn, green buckwheat, plantain.
- Second group—5 corn, green buckwheat, plantain.
- Third group—2 rice, corn, plantain; 7 rice, green buckwheat, chickpea flour; 8 rice, chickpea, plantain.
- Fourth group—4 amaranth flour, chickpea, corn flour; 6 amaranth flour, green buckwheat, corn flour.

The first group encompasses samples 1, 3, and 9, sharing a common composition of rice, green buckwheat, and plantain. These bread samples, belonging to the same cluster, likely exhibit similar organoleptic characteristics, as indicated by the results in Table 7. The second group, represented by sample 5, stands out due to its combination of corn, green buckwheat, and plantain. This unique composition places it in a separate cluster with distinct qualities.

The third group consists of samples 2, 7, and 8, incorporating variations of rice, corn, green buckwheat, and chickpea flour. These samples form a cluster based on their proximity in the feature space defined by the cluster analysis. The fourth group includes samples 4 and 6, utilizing amaranth flour, chickpea, and corn flour, showcasing a distinct cluster with its characteristic attributes.

The cluster analysis for bread types is not only a statistical classification but also a practical representation of how the choice of flours influences the final product. The identified clusters likely correspond to variations in the appearance, color, porosity, and overall organoleptic properties of the bread samples. The subsequent organoleptic evaluations and analyses presented in the paper further support the meaningfulness of these clusters.

The correlation between flour and bread clusters underscores the importance of selecting specific flour combinations to achieve desired qualities in the final product. The paper’s exploration of gluten content, amino acid compositions, and vitamin content in the bread samples is likely linked to these distinct clusters, demonstrating the impact of flour choices on both sensory and nutritional aspects of gluten-free bread.

Among these breads, sample No. 5 made from corn and green buckwheat flour, plantain in a ratio of 40:40:20 had the best indicators. This bread meets all the requirements of respondents. The bread was soft, porous, elastic, light brown in color, kneaded without separation, and without foreign odors and taste. 

### 3.4. Gluten Content in Bread

Table 9 below shows the gluten content in four different types of breads depending on the method of measurement.

According to the results of the study, the gluten content in all four samples did not exceed 0.6 mg/kg, which means that these products can be included in the list of gluten-free products [75].

### 3.5. Amino Acids and Vitamin Composition of the Bread

**Figure A1:**
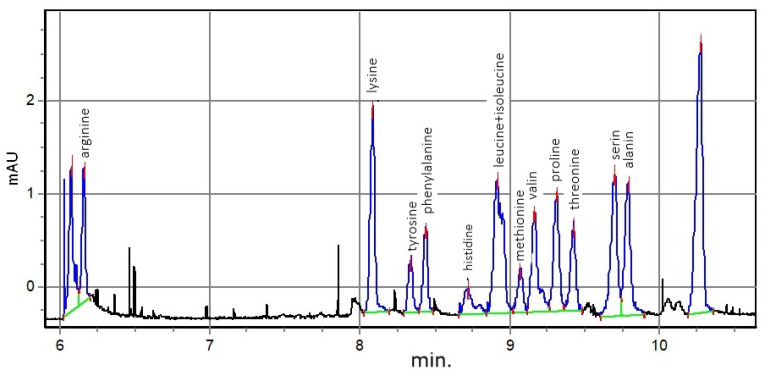
Amino acid content in bread No. 5.

The amino acid compositions of bread No. 5 are shown in Table 10 and Table A1 and Figure A1.

According to the results of the study on the content of amino acids in gluten-free breads, the most interchangeable amino acid among amino acids is glycine 0.458 ± 0.156;, among non-exchangeable amino acids, valine is 0.336 ± 0.134%, the smallest non-exchangeable amino acid, methionine, is 0.150 ± 0.051%, and tyrosine is 0.198 ± 0.060%.

**Figure A2:**
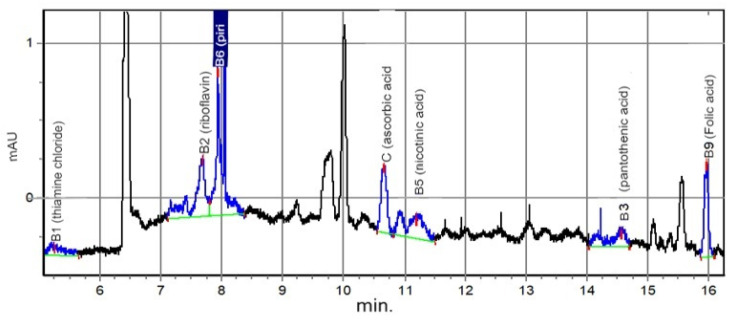
Amount of vitamins in bread No. 5.

The vitamin composition of bread No. 5 is shown in Table 11 and Table A2 and Figure A2.

According to the results of the study, among the water-soluble vitamins in baked bread No. 5, the largest amount per 100 g of vitamin from group B is found in B5 (pantothenic acid) 0.688 ± 0.138 mg, and the smallest amount is Vitamin B9 (folic acid) 0.081 ± 0.016 mg. The increased content of vitamins and dietary fiber in the finished product improved the nutritional profile of the bread. Comparing the results with the research work of Yuthana et al., it can be seen that the content of vitamins in gluten-free bread is high, and the indicators meet all standards [76].

## 4. Conclusions

The research aimed to develop an advanced technology for producing gluten-free bread by meticulously analyzing various gluten-free flours. By experimenting with different combinations of ingredients, the technological process was fine-tuned to achieve a successful recipe for gluten-free bread containing corn, green buckwheat, and plantain flour in a ratio of 40:40:20. The key discovery was a unique gluten-free blend comprising cereal-based and pseudo-cereal-based flours, which remarkably enhanced the texture and taste of the gluten-free bread while maintaining a gluten content below 3 ppm. As a result, the gluten-free bread exhibited characteristics closely resembling traditional wheat bread, boasting excellent color, aroma, softness, and full compatibility with gluten-free diets. Moreover, the study delved into the nutritional composition of gluten-free bread. While these results were promising, further investigation is essential to explore the potential effects of gluten-free fermentation properties.

In conclusion, the research was successful, elevating the technology for gluten-free bread production through meticulous analysis of gluten-free flours. The valuable findings contribute significantly to the advancement of gluten-free bread products, offering essential insights to manufacturers and researchers. Future studies should focus on investigating the intricate interplay between different compositions of gluten-free flours or alternative ingredients, thereby optimizing the gluten-free bread recipe further. These ongoing endeavors will undoubtedly deepen our understanding and expand the scope of possibilities for creating top-tier, high-quality gluten-free bread products.

The scientific value of the paper lies in its contribution to the development of high-quality gluten-free bread, the novel approach of combining flours, and the comprehensive analysis of various aspects of the bread-making process. It provides practical insights for both the food industry and researchers interested in improving gluten-free products.

## 5. Patents

Utarova Nazira Bakytzhanova, Kakimov Mukhtarbek Mukanovich, Nurtayeva Ainur Bolatbekovna, and Akshoraeva Gauhar Dyusengalievna. Method of production of gluten-free bread. The utility model was granted patent No. 7039, issued on 10 March 2022.

## Figures and Tables

**Table 1:** Health implications of the consumption of gluten-free bread.

Health Implication	Description
Celiac Disease Management	Essential for individuals with celiac disease to prevent autoimmune reactions triggered by gluten.
Non-Celiac Gluten Sensitivity	May provide relief for individuals with non-celiac gluten sensitivity, although more research is needed.
Nutritional Concerns	Potential for nutrient deficiencies, especially in fiber, iron, and B vitamins if not fortified.
Weight Management	Gluten-free bread can be higher in calories and less satiating, potentially impacting weight management.
Gastrointestinal Health	May help improve gastrointestinal symptoms in those with gluten-related digestive issues.
Diabetes Management	Gluten-free bread’s glycemic index can vary, impacting blood sugar control for those with diabetes.
Bone Health	Possible reduction in calcium intake if not fortified, affecting bone health, especially in children.

Source: Author’s analysis on the basis of: [14,19,20,22,23,24,25,26,27,28,29,30,31,32,33,34,35].

**Table 2:** Advantages and problems of gluten-free bread consumption.

Aspect	Adventages	Problems
Celiac Disease	Safe for individuals with celiac disease	Limited gluten-free options in some areas
Non-Celiac Sensitivity	May alleviate symptoms in sensitive individuals	Limited scientific understanding of sensitivity
Allergen-Free Options	Suitable for those with wheat allergies	Higher cost compared to wheat-based bread
Increased Awareness	Raised awareness of gluten-related disorders	May lead to unnecessary dietary restrictions
Diverse Ingredients	Encourages variety in diet	Texture and taste differences from wheat bread
Gastrointestinal Relief	Can reduce digestive discomfort	Availability and affordability of options
Customizable Nutrition	Some products enriched with nutrients	May be lower in fiber and essential nutrients
Culinary Creativity	Promotes innovative gluten-free recipes	May require special baking skills and ingredients
Dietary Flexibility	Expands dietary options for those without gluten intolerance	Potential for unbalanced diets

Source: Author’s analysis on the basis of: [21,22,23,27,28,29,30,31,32,33,34,35,36,37,38,39,40].

**Table 3:** Selected parameters of the flours used for the tests.

	Desired Quantity, %	Component Property
Corn	40–50	It is rich in vitamins: A, C, B3, E, D, K, Group B, and also contains valuable minerals: K, Ca, P, Fe, and Mg, as well as trace elements—Ni and Cu. Corn protein contains important amino acids—tryptophan and lysine. Regular consumption of corn can reduce the risk of stroke, diabetes, and cardiovascular disease [55].
Green buckwheat	40	It contains trace elements necessary for the body: Fe, P, Cu, Mg, K, Zn, Si, S, Mn, and many other trace elements. It has a high biological value and is a gluten-free raw material due to a high content of lysine, which has an amino acid digestibility coefficient of proteins in its composition of 99.45% [56].
Rice	40–50	It contains a large amount of Cu, due to which blood composition is normalized. Rice flour contains several vitamins and minerals that help reduce the amount of sugar in the blood, reduce excess fluid, salt, and toxins in the body, improve heart function, and quickly restore the body after diseases and physical exertion [57].
Chickpea	40–50	It contains a low glycemic index and a high level of protein and natural fiber; it has unsaturated and saturated fatty acids, vitamins B, A, K, PP, E, C; beta-carotene, Mn, K, Na, Mg, Se, Ca, Zn, Cl, Fe, I, P, S, Mo, Pb, V, Si, Ti [58].
Amaranth	40–50	It is rich in high-quality protein and contains essential amino acids and fats, including 50% polyunsaturated omega-6 fatty acids acid, and it contains a significant amount of vitamins E, A, B1, B2, Choline (B4), C, and D [59].
Plantain	10–20	It is known for its healing properties, and it is a rich source of dietary fiber. It is the source of important macro and microelements, which are so necessary for the human body, and as recent studies have shown, it is an important dietary component that affects the gastrointestinal tract, cardiovascular system, metabolism, and immunity [11], improving intestinal barrier function and microbial composition.

**Table 4:** Relations of raw materials for the preparation of gluten-free bread.

Sample No.	Sample Flours	Ratio
1	rice, green buckwheat, plantain	50:40:10
2	rice, corn, plantain	50:40:10
3	rice, green buckwheat, plantain	40:40:20
4	amaranth flour, chickpea, corn flour	50:40:10
5	corn, green buckwheat, plantain	40:40:20
6	amaranth flour, green buckwheat, corn flour	40:40:20
7	rice, green buckwheat, chickpea flour	50:40:10
8	rice, chickpea, plantain	50:40:10
9	corn, green buckwheat, plantain	50:40:10

**Table 5:** Organoleptic indicators of flours (*n* = 20).

Type of Flour	Name of Indicators	Characteristics and Norms [67,68,69]	The Result	Score
	Appearance	Homogeneous, bulk product with small shell particles	----	8.75 ± 1.37
	Color	White or yellow	Light yellow	9.10 ± 1.29
Corn	Smell	Typical of cornmeal, odorless, non-moldy	Characteristic of corn flour, no foreign smell, no mold.	9.10 ± 0.97
	Taste	Characteristic of corn flour, does not have sour, bitter, or other taste	No foreign taste, not sour, not bitter	8.35 ± 1.31
			Average	8.82
	Appearance	Whitish, homogeneous product with small particles of flakes	----	9.15 ± 0.81
	Color	Light brown, creamy, with a brownish-grey tint	Light brown	9.20 ± 0.77
Green buckwheat	Smell	Characteristic of green buckwheat flour, odorless, without mold	Characteristic of green buckwheat flour, no foreign smell, no mold.	9.10 ± 0.85
	Taste	Characteristic of green buckwheat flour, there is no sour, bitter, or other taste	No foreign taste, not sour, not bitter	9.25 ± 0.85
			Average	9.17
	Appearance	Homogeneous, bulk product with small shell particles	----	8.30 ± 0.73
	Color	White with white, cream, or yellowish tints	White	8.00 ± 0.92
Rice	Smell	Characteristic of rice flour, odorless, without mold	Characteristic of rice flour, no foreign smell, no mold	7.65 ± 0.81
	Taste	Characteristic of rice flour, no sour, bitter, or other taste	No foreign taste, not sour, not bitter	7.90 ± 0.85
			Average	7.96
	Appearance	Homogeneous, bulk product with small shell particles	----	8.20 ± 0.89
	Color	Yellow with shades of grey	White-grey with shades of grey	8.25 ± 0.97
Chickpea	Smell	Characteristic of chickpeas, without foreign odors, without mold	Characteristic of chickpeas, no foreign smell, no mold	8.05 ± 0.76
	Taste	Characteristic of chickpeas, without sour, bitter, and other taste	No foreign taste, not sour, not bitter	7.30 ± 0.92
			Average	7.95
	Appearance	Homogeneous, bulk product with small shell particles	----	7.85 ± 1.23
	Color	White-brown with a gray tint	White-brown with a gray tint	7.52 ± 0.83
Amaranth	Smell	Characteristic of amaranth, without foreign odors, without mold	Characteristic of amaranth, no foreign smell, no mold	7.43 ± 0.83
	Taste	Amaranth has no characteristic, sour, bitter, and other taste	No foreign taste, not sour, not bitter	7.80 ± 1.01
			Average	7.61
	Appearance	Homogeneous, bulk product with small shell particles	----	8.50 ± 0.83
	Color	White-grey with shades of grey	White-grey with shades of grey	8.60 ± 0.75
Plantain	Smell	Plantain-specific, without foreign odors, without mold	Characteristic of plantain, no foreign smell, no mold	9.30 ± 0.73
	Taste	Plantain has no characteristic, sour, bitter, and other taste	No foreign taste, not sour, not bitter	8.90 ± 0.97
			Average	8.83

**Table 6:** Physico-chemical indicators of green buckwheat, rice, amaranth, chickpea, corn meal, and plantain flours (*n* = 1).

	Max. Moisture Content Acc. to GOST 9404-88	Moisture, %	Fiber, %	Bulk Density, kg/m^3^	Ash Content on Dry Basis, %
Corn	15.0	8.23	7.3	544	0.3060
Green buckwheat	12.0	7.85	10.6	539	0.4264
Rice	12.0	9.11	2.4	538	0.3689
Chickpea	12.0	7.91	3.5	423	0.3005
Amaranth	15.0	8.32	4.7	613	0.4120
Plantain	15.0	7.18	11.9	539	0.8295

**Table 7:** Results of surveys conducted for the organoleptic evaluation of bread.

No.	Flours	Appearance	Color	Smell	Porosity	Taste	Average Score	Place in the Ranking
1	rice, green buckwheat, plantain	4.11 ± 0.61	3.81 ± 0.58	4.28 ± 0.65	3.58 ± 0.68	4.40 ± 0.51	4.04	**3**
2	rice, corn, plantain	3.50 ± 0.64	4.05 ± 0.65	3.00 ± 0.44	3.40 ± 0.56	3.45 ± 0.62	3.48	7
3	rice, green buckwheat, plantain	4.22 ± 0.44	3.94 ± 0.46	4.28 ± 0.48	4.17 ± 0.51	4.17 ± 0.62	4.16	**2**
4	amaranth flour, chickpea, corn flour	3.33 ± 0.52	3.06 ± 0.58	2.56 ± 0.63	3.33 ± 0.59	2.94 ± 0.64	3.04	9
5	corn, green buckwheat, plantain	4.44 ± 0.57	4.11 ± 0.63	4.56 ± 0.56	4.33 ± 0.51	4.61 ± 0.50	4.41	**1**
6	amaranth flour, green buckwheat, corn flour	3.28 ± 0.40	3.11 ± 0.38	2.83 ± 0.33	3.06 ± 0.24	2.94 ± 0.24	3.04	8
7	rice, green buckwheat, chickpea flour	3.94 ± 0.65	3.72 ± 0.62	4.06 ± 0.50	3.44 ± 0.56	3.44 ± 0.62	3.72	5
8	rice, chickpea, plantain	3.67 ± 0.61	3.78 ± 0.71	4.00 ± 0.65	3.83 ± 0.56	3.28 ± 0.46	3.71	6
9	corn, green buckwheat, plantain	4.00 ± 0.45	3.94 ± 0.59	4.50 ± 0.73	3.67 ± 0.65	4.00 ± 0.69	4.02	**4**

The results are displayed as the mean ± the standard deviation (*n* = 18).

**Table 8:** Organoleptic characteristics of bread samples.

Sample No.	Appearance	Color	Color in the CIELab Scale			Crumb Condition	Porosity	Taste and Smell
			L	a	b			
1	The correct shape, smooth, without big cracks	Light brown	58	7	30	Baked, elastic, wet	Thin-walled, developed, voids are small	No foreign taste or smell, it is characteristic of this species product
3	The correct shape, smooth, there are cracks	Light brown	56	7	37	Baked, elastic, not damp to the touch	Uneven, with large pores in the crumb	No foreign taste or smell, it is characteristic of this species product
5	The correct shape, smooth	Light brown	58	11	41	Baked, elastic, not damp to the touch	Uniform, porous, with small pores	No foreign taste or smell, it is characteristic of this species product
9	The correct shape, no large cracks	Dark brown	57	3	30	Baked, elastic, slightly damp	Uniform, without pores, there are a bit of pores	No foreign taste or smell, it is characteristic of this species product

**Table 9:** Gluten content in gluten-free bread (*n* = 3).

Sample	Gluten Concentration, (mg/kg)		
	RIDESCREEN (R5 ELISA)	Veratox (R5 ELISA)	AgraQuant (G12 ELISA)
1	0.60 ± 0.08	0.52 ± 0.03	0.6 ± 0.08
3	0.53 ± 0.04	0.53 ± 0.08	0.42 ± 0.03
5	0.30 ± 0.01	0.21 ± 0.01	0.22 ± 0.01
9	0.29 ± 0.02	0.22 ± 0.01	0.31 ± 0.03

**Table 10:** Amino acid content in bread No. 5.

No.	Component	Mass Fraction of Amino Acids, %
1	Arginine	0.375 ± 0.150
2	Lysine	0.245 ± 0.083
3	Tyrosine	0.173 ± 0.052
4	Phenylalanine	0.274 ± 0.082
5	Histidine	0.173 ± 0.087
6	Methionine	0.120 ± 0.041
7	Leucine + isoleucine	0.346 ± 0.090
8	Valin	0.274 ± 0.110
9	Proline	0.346 ± 0.090
10	Threonine	0.188 ± 0.075
11	Serin	0.260 ± 0.068
12	Alanin	0.274 ± 0.071
13	Glycine	0.260 ± 0.088

**Table 11:** Amount of vitamins in gluten-free bread No. 5.

No.	Component	Conc., mg/100 g.
1	B1 (thiamine chloride)	0.130 ± 0.026
2	B2 (riboflavin)	0.369 ± 0.155
3	B6 (pyridoxine)	0.418 ± 0.084
4	C (Ascorbic Acid)	1.352 ± 0.460
5	B5 (pantothenic acid)	0.688 ± 0.138
6	B3 (nicotinic acid)	0.106 ± 0.019
7	B9 (folic acid)	0.081 ± 0.016

## Data Availability

Data is contained within the article.

## Appendix A

**Table A1:** The details of amino acid content in bread No. 5.

No.	Component	Height, m	The Beginning Process, min	End, min	Volume, cm^3^	Conc, mg/L
1	Arginine	1.333	6.138	6.205	22.58	26.0
2	Lysine	1.651	8.055	8.208	35.91	17.0
3	Tyrosine	0.498	8.322	8.417	11.65	12.0
4	Phenylalanine	1.191	8.417	8.517	20.04	19.0
5	Histidine	0.242	8.693	8.885	12.81	12.0
6	Methionine	0.392	9.065	9.148	9.99	8.30
7	Leucine + isoleucine	1.680	8.885	9.065	64.55	24.0
8	Valin	1.070	9.148	9.298	29.02	19.0
9	Proline	1.484	9.298	9.415	38.84	24.0
10	Threonine	0.814	9.415	9.507	20.68	13.0
11	Serin	1.122	9.658	9.778	33.64	18.0
12	Alanin	1.619	9.778	9.903	44.69	19.0
13	Glycine	1.841	10.232	10.382	53.42	18.0

**Table A2:** The details of the amount of vitamins in gluten-free bread No. 5.

No.	Component	Height, m	The Beginning of the Process, min	End, min	Volume, cm^3^	Conc., mg/L
1	B1 (thiamine chloride)	0.074	5.045	5.658	13.82	0.0053
2	B2 (riboflavin)	0.379	7.112	7.805	47.45	0.015
3	B6 (pyridoxine)	0.941	7.805	8.372	74.31	0.017
4	C (Ascorbic Acid)	0.441	10.540	10.817	30.21	0.055
5	B5 (pantothenic acid)	0.143	10.817	11.495	36.9	0.028
6	B3 (nicotinic acid)	0.104	14.033	14.697	22.4	0.0043
7	B9 (folic acid)	0.610	15.862	16.075	29.51	0.0033
